# Ovulatory Signal-Driven H3K4me3 and H3K27ac Remodeling in Mural Granulosa Cells Orchestrates Oocyte Maturation and Ovulation

**DOI:** 10.3390/cells15010034

**Published:** 2025-12-24

**Authors:** Furui Wang, Wenjing Wang, Shuai Zhang, Yinjuan Wang, Ruimen Zhang, Lei An, Jianhui Tian, Guangyin Xi

**Affiliations:** 1 College of Animal Science and Technology, Frontiers Science Center for Molecular Design Breeding (MOE), China Agricultural University, Beijing 100193, China; wangfuruifreedom@163.com (F.W.); wwjlilili@163.com (W.W.); isshuai.zhang@foxmail.com (S.Z.); wangyinjuan@cau.edu.cn (Y.W.); anleim@cau.edu.cn (L.A.); tianjh@cau.edu.cn (J.T.); 2 Key Laboratory of Efficient Utilization of Non-Grain Feed Resources (Co-Construction by Ministry and Province), Ministry of Agriculture and Rural Affairs, Shandong Provincial Key Laboratory of Animal Nutrition and Efficient Feeding, Department of Animal Science, Shandong Agricultural University, Taian 271017, China; 3 College of Animal Science and Technology, Guangxi University, Nanning 530004, China; 20230099@gxu.edu.cn

**Keywords:** luteinizing hormone, histone modification, mural granulosa cells, ovulation, oocyte maturation, H3K4me3, H3K27ac

## Abstract

**Highlights:**

**What are the main findings?**

- During ovulatory signal-induced ovulation, a precisely timed transcriptional reprogramming in mural granulosa cells (MGCs) is evoked, with distinct temporal waves that reflect the progression from oocyte maturation to ovulatory response.
- H3K4me3 and H3K27ac represent two core histone-modification programs underlying LH-induced transcription. H3K4me3 activates target gene expression primarily by enriching in gene promoter regions; H3K27ac regulates MGCs transcription by establishing enhancers, especially SEs.

**What are the implications of the main findings?**

- Our study demonstrates that super-enhancers (SEs) constitute a key regulatory axis through which the ovulatory signal amplifies gene activation in MGCs.
- The integration of temporal transcriptional changes with dynamic promoter and SE remodeling provides a framework for how gonadotropins coordinate MGC function through chromatin architecture, offering new mechanistic insights into the epigenetic orchestration of follicular development.

**Abstract:**

Ovulation and granulosa cell luteinization are induced by ovulatory signals, including luteinizing hormone (LH) and human chorionic gonadotropin (hCG). Histone modifications enable rapid, signal-responsive transcriptional reprogramming. However, the effects of LH/hCG-induced histone modification changes on the mural granulosa cells (MGCs) function remain to be fully elucidated. By mining public datasets we integrated transcriptomic and histone-modification profiles of MGCs across the ovulatory interval and tracked LH/hCG-driven gene expression at three time points (0, 4, and 12 h after-hCG). During oocyte maturation, the 4 h LH-surge constitutes a critical window for meiotic resumption, during which many genes display rapid transcriptional changes followed by a return to baseline levels. Early-response genes are enriched for cell locomotion, inflammatory responses, the activation of signaling pathways, and histone modifications. Furthermore, LH/hCG-induced transcriptome remodeling is highly correlated with dynamic gains or losses of H3K4me3 and H3K27ac. Notably, we discovered for the first time that H3K27ac marks super-enhancers (SEs) that regulate LH/hCG-induced transcriptional activation in MGCs. Finally, through complementary in vitro and in vivo pharmacological inhibition, we demonstrate that LH/hCG governs oocyte maturation and ovulation by reshaping the MGC transcriptome via H3K4me3- and H3K27ac-dependent chromatin remodeling. In summary, our study advances the understanding of how gonadotropins regulate MGC function and oocyte maturation through histone-modification-mediated transcriptional control.

## 1. Introduction

Proper folliculogenesis is a prerequisite for the generation of mature, fertilization-competent oocytes. During the secondary follicle stage, granulosa cells (GCs) differentiate into two types: mural granulosa cells (MGCs) and cumulus cells (CCs). MGCs, which are adjacent to the follicular basement membrane, form the inner layer of the follicle wall [1,2]. Luteinizing hormone (LH), a key gonadotropin in the hypothalamus–pituitary–gonadal axis, is specifically recognized by its receptor, LH/chorionic gonadotropin receptor (LHCGR), expressed on MGCs within the follicle [3]. Prior to the ovulation of the dominant follicle, LH levels peak in a phenomenon known as the LH-surge [4]. In vivo, the preovulatory LH-surge triggers follicular maturation and ovulation. In experimental mouse models, this physiological LH-surge is commonly mimicked by the administration of human chorionic gonadotropin (hCG), which activates the same LHCGR-mediated signaling pathway [5,6]

Upon LH stimulation, the secretion of proteases in the follicular fluid increases, leading to the degradation of the follicular wall of the preovulatory follicle. The oocyte and the surrounding CCs form the cumulus–oocyte complex (COC), which is subsequently released into the oviduct to complete ovulation [7]. LH also activates many downstream signaling pathways, including the ERK1/2, to stimulate meiotic resumption in the oocyte, accompanied by germinal vesicle breakdown (GVBD) and extrusion of the first polar body, yielding a mature metaphase II (MII) oocyte [8,9]. Meanwhile, LH drives extensive tissue remodeling in the follicular wall, including epidermal cell shedding, connective tissue-degradation, vasoconstriction, etc. [10,11,12], which together create an apical rupture site for COC release [13]. The LH-activated ERK1/2 pathway also plays an important role in follicle rupture [14]. Dysregulation of these pathways in MGCs is usually associated with polycystic ovary syndrome (PCOS) and ovulatory dysfunction. In addition, genes involved in CC expansion, such as *Ptgs2*, *Tnfaip6*, *Ptx3*, and *Pgr*, are transiently up-regulated following LH stimulation [15,16].

In addition to promoting ovulation, LH/hCG induces the transformation of MGCs into luteal cells, forming the corpus luteum (CL) [17]. This transition accompanies a functional switch from estrogen synthesis to progesterone production, culminating in luteinization [18]. The cellular identities of MGCs and luteal cells are largely defined by the expression of steroidogenic enzymes, including *Star*, *Cyp11a1*, *Hsd3b*, and *Hsd17b* [19,20]. Although the precise timing of luteinization initiation and completion remains unclear, evidence suggests that the differentiation of MGCs into luteal cells spans more than 12 h [21,22]. Similarly to ovulation, the process of luteinization is also accompanied by extensive tissue remodeling, including cell migration and angiogenesis. The formation of a dense capillary network facilitates the uptake of nutrients such as cholesterol, enabling steroidogenesis, and this process is regulated by vascular endothelial growth factor (VEGF) [23,24].

Gonadotropins orchestrate a reprogramming of the epigenetic landscape and chromatin accessibility within GCs, thereby modulating their transcriptional output [25,26,27,28]. H3K4me3 and H3K27ac, two well-established histone marks associated with transcriptional activation, facilitate rapid gene-expression changes by modulating chromatin accessibility and transcription factor binding. H3K4me3 preferentially accumulates near gene promoters, whereas H3K27ac marks active promoters and distal enhancers that drive robust transcription [29,30,31,32]. Research has demonstrated that FSH can markedly enhance gene expression in ovarian GCs by altering the H3K4me3 enrichment at loci such as *Lhcgr*, *Cyp19a1*, and *Nppc* [33]. LH signaling similarly increases H3K4me3 enrichment at the promoter regions of steroidogenic genes, including *Star*, *Hsd3b,* and *Cyp11a1* [25,28,34]. Moreover, LH induces broad histone deacetylation, resets the chromatin structure, and triggers H3K27ac reprogramming during the luteinization of MGCs [35]. LH also down-regulates *Hdac3* levels, promotes the establishment of histone H3K14ac, and generates an activated chromatin state that enhances Sp1, thereby stimulating *Areg* transcription [4,36].

In the present study, leveraging public transcriptome datasets, we tracked the transcriptional response of MGCs to LH/hCG at three pivotal ovulation-induction time points (0, 4, and 12 h). KEGG (Kyoto Encyclopedia of Genes and Genomes) and GO (Gene Ontology) analyses delineated the transient and dynamic reprogramming of gene expression and associated cellular processes induced by LH/hCG. The integration of RNA-seq with H3K4me3 and H3K27ac ChIP-seq maps revealed the multi-layered transcriptional regulation downstream of ovulatory signaling. To interrogate enhancer-mediated gene expression regulation, Rank Ordering of Super-Enhancers (ROSE) was employed to identify LH/hCG-induced H3K27ac super-enhancers (SEs). Finally, through complementary in vitro culture and in vivo pharmacological inhibition, we demonstrated that LH/hCG reshapes the MGC transcriptome through coordinated H3K4me3 and H3K27ac remodeling, thereby directing oocyte maturation and ovulation. Collectively, our study resolves the temporal dynamics of LH/hCG-induced transcriptional programs in MGCs, establishes histone modifications—particularly promoter-associated H3K4me3 and SE-associated H3K27ac—as key regulators of this process, and provides mechanistic insight and potential epigenetic targets for improving oocyte-maturation strategies and clinical applications.

## 2. Materials and Methods

### 2.1. Animals

ICR (CD-1) female mice aged 3 wk used in the present study were purchased from SiPaiFu Biotechnology Co., Ltd. (Beijing, China) and were housed under controlled conditions (temperature: 20–25 °C, 12 h light/dark cycle) in an IVC feeding isolation system for experimental animals. They had unrestricted access to food and water.

### 2.2. Collection of Mouse MGCs

Immature 3 wk mice were intraperitoneally injected with 5 IU PMSG (Sansheng Pharmaceutical, Ningbo, China) to stimulate follicle development. After 48 h, 5 IU hCG (Sansheng Pharmaceutical, Ningbo, China) was injected to stimulate ovulation. The ovaries were obtained 0 h, 4 h, or 12 h after hCG injection. After removing excess adipose tissue, the ovaries were transferred into Medium 199 (Gibco, Carlsbad, CA, USA). Under a stereomicroscope, large antral follicles were punctured with a syringe to release the follicle contents, and the COCs were removed by oral pipette. The remaining MGCs were washed twice with pre-cooled PBS, centrifuged, and stored at −80 °C.

### 2.3. In Vitro Culture of Mouse MGCs

Mice were intraperitoneally injected with 5 IU PMSG for 48 h before being euthanized. The mouse ovaries were isolated, and the adipose tissue was removed and placed in MGC-A solution, which was prepared by mixing 500 μL of 100× Penicillin–Streptomycin (Gibco, Carlsbad, CA, USA) and 5 mL of FBS (VivaCell, Denzlingen, Germany), and bringing the volume up to 50 mL with DMEM/F-12, HEPES (Gibco, Carlsbad, CA, USA). Under a stereomicroscope, the follicles were punctured with a syringe to release the contents. After collection, the MGCs were centrifuged at 2000 rpm for 5 min, washed twice with PBS, resuspended in MGC-A solution, and plated. After 4 h, they were washed with PBS, and then cultured with MGC-B solution, composed of 100× Penicillin-Streptomycin 500 μL, FBS 5 mL, 0.3 μg/μL E2 5 μL, DMEM/F-12, HEPES to 50 mL) in a 37 °C incubator with 5% CO_2_ for 24 h overnight. Before cell treatment, cells were washed again with PBS to remove non-adherent cells, and then replaced with MGC-C solution (100× Penicillin-Streptomycin 500 μL, FBS 5 mL, 0.3 μg/μL E2 5 μL, 4 ng/μL Epidermal growth factor (EGF) 62.5 μL, DMEM/F-12, HEPES to 50 mL), and other treatment solutions. EGF exerts its effects by binding to the EGF receptor (EGFR); the rapid expansion and propagation of LH signals within the preovulatory follicle is achieved precisely by activating EGFR [37,38]. Because EGF is more stable in vitro than LH and can mimic the effects of LH, it was used in place of LH in the in vitro cell experiments of this study.

### 2.4. Inhibitor Treatment

We used JQ-1 (an inhibitor of the H3K27ac modification enzyme BRD4) [39,40] and Bcl-121 (an inhibitor of the H3K4me3 modification enzyme SMYD3) [41] to interfere with H3K27ac and H3K4me3 modifications to explore the effects of these two histone modifications on LH/hCG-induced MGC gene expression, follicle ovulation rate, and oocyte maturation. JQ-1 and Bcl-121, both from TargetMol (T5443 and T5322, Shanghai, China), were fully dissolved in DMSO and stored in a −80 °C refrigerator. The stock solution was added to the MGCs culture medium to make the working concentration of JQ-1 and Bcl-121 200 nM, and the inhibitors were pretreated for 1 h before EGF induction. In the in vivo mouse inhibitor injection model, JQ-1 and Bcl-121 were diluted to 1 mM and 4 mM with physiological saline, and the injection doses were 0.3 mL/kg and 0.15 mL/kg (120.264 mg/kg and 204.132 mg/kg), respectively, and the inhibitors were pretreated for 4 h before hCG induction.

### 2.5. RNA Extraction and Quantitative Reverse Transcription PCR (RT-qPCR)

Total RNA was extracted from MGCs using the TRIzol reagent (Thermo Fisher Scientific, Waltham, MA, USA) according to the manufacturer’s protocol. Then, 1 μg RNA was reverse transcribed into cDNA by 4×HiScript II qRT SuperMix II (Vazyme Biotech Co., Ltd., Nanjing, China). Then, qPCR reactions were performed using the SYBR Green master mix (Vazyme Biotech Co., Ltd., Nanjing, China) on CFX96 Touch Real-Time PCR Detection System (Bio-Rad Laboratories, Hercules, CA, USA). All assays were performed at least 3 times. Primers used in this study are listed in Supplementary Table S1. Relative expression was determined from the threshold cycle (CT), normalized to the reference genes [42].

### 2.6. Quality Assessment and Analysis of MGCs Transcriptome Data

The mouse MGC transcriptome sequencing data used in this study was derived from the public database GEO (https://www.ncbi.nlm.nih.gov/geo/ (accessed on 3 October 2022)), and the original data project number is GSE167939. The specific steps of sample collection were as shown in the article [43]. Each time point contains three biological replicates, and all replicates available in the original studies were included in the analysis.

Trim Galore software 0.6.7 was used to remove adapters and perform quality control; Hisat2 software 2.2.1 was used to construct the mouse genome mm10 index; Subread software 2.0.2 was used to quantify the aligned data, and the mouse reference genome annotation file was downloaded from the Ensemble database. The featureCounts command was used to quantify the reads. Then the count was converted to transcripts per kilobase per million mapped reads (TPM) for subsequent analysis, only retaining genes with TPM > 0.5 in at least half of the biological replicates for subsequent analysis. Difference analysis was performed using the R package Deseq2 1.30.1. After normalizing the number of gene counts in each sample, the difference fold was calculated, and the difference significance test was performed. The screening criteria for differentially expressed genes (DEGs) are |Fold Change| > 2 and *p*-adj < 0.05. The R package CluterProfiler was used to perform GO and KEGG enrichment analysis on DEGs, and describe the corresponding gene functions; Short Time-series Expression Miner (STEM) analyses were conducted to explore the expression profiles of DEGs during different stages after hCG injection.

Among the samples, the proportion of genes with expression levels 0.5 < TPM < 100 was the highest, and the proportion of low-expression genes (0 < TPM < 0.5) and high-expression genes (100 < TPM) was relatively small, indicating that each sample had good sequencing depth and quality; a considerable number of low-expression genes were detected, and the distribution between biological replications was also highly similar (Figure S1A). To prevent the impact of low-expression genes on our analysis, we used a threshold of TPM > 0.5 in at least one sample as our criterion, filtering out low-expression genes and categorizing the remaining genes. Subsequently, we selected protein-coding genes for further analysis (Figure S1B). Following hCG induction, the transcriptional states varied at different time points, and the differences between sample groups were significant, allowing for an effective division into 3 developmental stages for subsequent differential analysis (Figure S1C,D).

### 2.7. CHIP-Seq Data Analysis

In this study, ChIP-seq profiles of mouse MGCs were taken from the public database GEO, original data accession GSE167938 and GSE165809. The two data sources have the same experimental method and are feasible for comparison. The 3 wk old mice were intraperitoneally injected with 5 IU PMSG to stimulate follicle development, and 48 h later, 5 IU hCG was injected to stimulate ovulation. The mice were euthanized at 0 h or 4 h after hCG injection, and the mouse ovaries were removed. After stripping off excess adipose tissue, large antral follicles were punctured with a syringe under a stereomicroscope to release MGCs for library construction. Each time point contains three biological replicates, and all replicates available in the original studies were included in the analysis.

Trim Galore software was also used to remove adapters and perform quality control; Bowtie2 software 2.4.5 was used to construct the mouse genome mm10 index, and then the alignment was performed. Samtools software 1.16.1 was used to remove PCR duplicates from the aligned samples with default parameters; MACS2 software 3.0.0 was used for peak detection; FindOverlapsOfPeaks 3.6.5 was used to perform differential fragment analysis on non-repeated samples; and R package Chipseeker 1.8.6 was used to annotate peak information.

### 2.8. Super-Enhancer Analysis

The ROSE 1.3.1 (https://bitbucket.org/young_computation/rose/src/master/ (accessed on 5 October 2022)) was used to perform the ROSE_main.py command on the H3K27ac ChIP-seq peak (callpeak) files; the ROSE algorithm was used to identify SEs and typical enhancers (TEs) in different samples [44,45]. The parameters were set to “−s12500 −t2000”, where the stitching distance of 12.5 kb defines the maximum distance allowed for merging adjacent enhancer peaks, and the TSS-exclusion window of 2 kb helps minimize promoter-associated signal contamination. Enhancer regions were stitched, ranked by H3K27ac signal density, and classified into SEs and TEs based on the inflection point of the ranking curve. Subsequently, the Python ROSE_geneMapper.py command was used to annotate enhancers to genes.

### 2.9. Immunohistochemical Staining

Ovarian tissues were collected, trimmed to remove excess fat, and fixed in 4% paraformaldehyde at 4 °C. Following fixation, the samples were dehydrated through a graded ethanol series using an automatic tissue processor (Leica Biosystems, Shanghai, China), cleared in xylene, and embedded in paraffin. Paraffin blocks were trimmed and sectioned at 5 μm thickness using a rotary microtome.

Freshly cut sections were floated on a 40–45 °C water bath solely for unfolding, mounted onto adhesive slides, and dried at 60 °C. Before staining, the slides were subjected to standard deparaffinization in xylene, followed by rehydration through graded alcohols to distilled water. The rehydrated slices were then microwaved for 20 min in a citric acid solution for antigen retrieval. Subsequently, the slices were incubated in a 3% hydrogen peroxide–methanol solution in the dark for 10 min, followed by blocking in a humidified box with a 1% bovine serum albumin (BSA, Sigma Chemicals Co., St. Louis, MO, USA) solution for 40 min at room temperature. The primary antibody, diluted in the blocking solution according to the specified ratio (Anti-H3K4me3, rabbit polyclonal, 1:100, ab8580; Anti-H3K27ac, rabbit polyclonal, 1:100; both of them from Abcam, ab4729, Cambridge, UK), was incubated overnight at 4 °C; and the secondary antibody, diluted and incubated at room temperature for 1 h in the dark (Goat anti-Rabbit IgG, Alexa Fluor™ 594, Invitrogen, A-11012, Carlsbad, CA, USA), was then applied. After staining the cell nucleus with DAPI (Vector Laboratories, Newark, CA, USA), the slides were sealed with resin and observed and photographed under a laser confocal microscope (Nikon Corp, Tokyo, Japan).

### 2.10. Statistical Analysis

Each experiment was conducted with a minimum of 3 replicates. Statistical analyses were performed using SPSS 22.0 (SPSS, Chicago, IL, USA). The data were analyzed using a one-way analysis of variance (ANOVA), and differences between groups were assessed using Tukey’s test for multiple means comparison. Wilcoxon rank sum test was also performed on sequencing data. All data are presented as means ± standard error of the mean (SEM).

## 3. Results

### 3.1. Transcriptome-Wide Changes Induced by LH/hCG in Mouse MGCs

**Figure 1:**
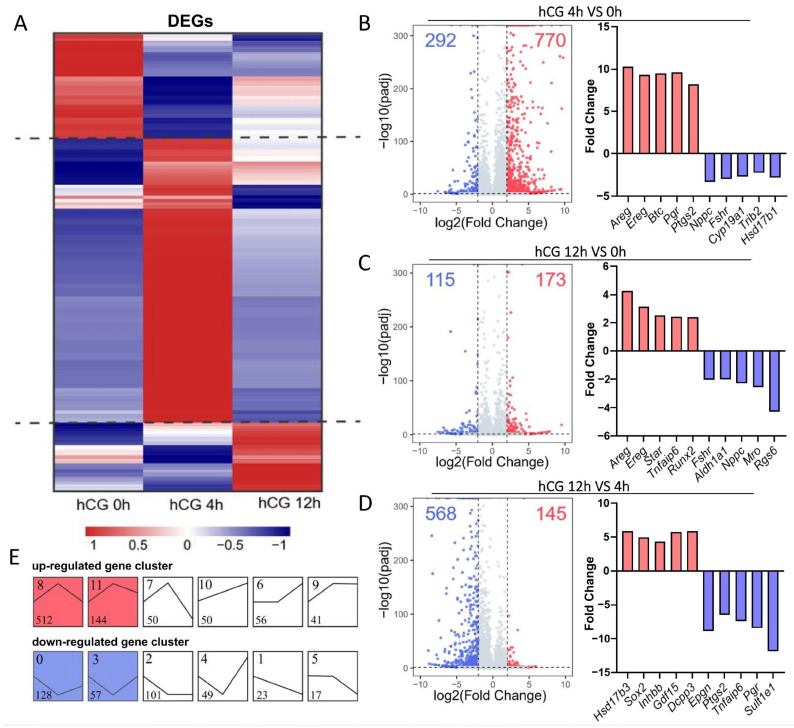
Characteristics of transcriptional dynamic changes in MGCs induced by human chorionic gonadotropin (hCG). (**A**) Heatmap showing DEGs across 0, 4, and 12 h after-hCG treatment. (**B**–**D**) Volcano plots showing the significance and log2 fold-change for genes detected in the RNA-seq analysis at each comparison (left), and fold change in the representative marker genes (right). (**E**) Temporal expression clusters identified by Short Time-series Expression Miner (STEM), each small panel represents a distinct expression cluster. The number in the upper left corner of each panel denotes the cluster ID, and the number in the lower left corner denotes the number of genes in that cluster. The black line represents the average expression trend of all genes within the cluster, only the colored panels indicate clusters with statistically significant expression patterns; white panels are not significant. In (**B**–**E**), red indicates significantly up-regulated genes and blue indicates significantly down-regulated genes.

To chart the transcriptomic response of mouse MGCs to the LH-surge, we analyzed gene expression at 0, 4, and 12 h after hCG (Figure 1A). At 4 h after hCG, 1062 DEGs were detected (770 up-regulated and 292 down-regulated; Figure 1B). As expected, EGF-like factor genes (*Areg*, *Ereg*, and *Btc)* and ovulation-related genes (*Ptgs2* and *Pgr)* were rapidly induced by hCG. The expression of follicle growth-related genes (*Nppc*, *Fshr*, and *Trib2*) and estrogen synthesis-related genes (*Cyp19a1* and *Hsd17b1*) was significantly down-regulated (Figure 1B).

The comparison of 12 h versus 0 h yielded 288 DEGs (173 up, 115 down; Figure 1C). The expression of ovulation-related genes (*Star*, *Tnfaip6*, and *Runx2*) was significantly up-regulated (Figure 1C). When 12 h was contrasted with 4 h, a total of 713 DEGs were found, including 145 significantly up-regulated genes and 568 significantly down-regulated genes (Figure 1D). Notably, *Pgr*, *Ptgs2*, *Epgn*, *Tnfaip6*, and *Sult1e1* showed a sharp decline, indicating that peak expression of key ovulatory genes occurs earlier and does not coincide with follicle rupture (Figure 1D).

Short Time-series Expression Miner (STEM) analysis clustered the DEGs into 12 temporal profiles (Figure 1E). The most significant patterns (blue and red modules) exhibited substantial changes at 4 h, followed by a return to baseline at 12 h, confirming that the primary wave of LH-responsive genes is transient and peaks well before ovulation, in full agreement with earlier reports [43].

### 3.2. Functional Enrichment Analysis of DEGs Induced by LH Signaling

**Figure 2:**
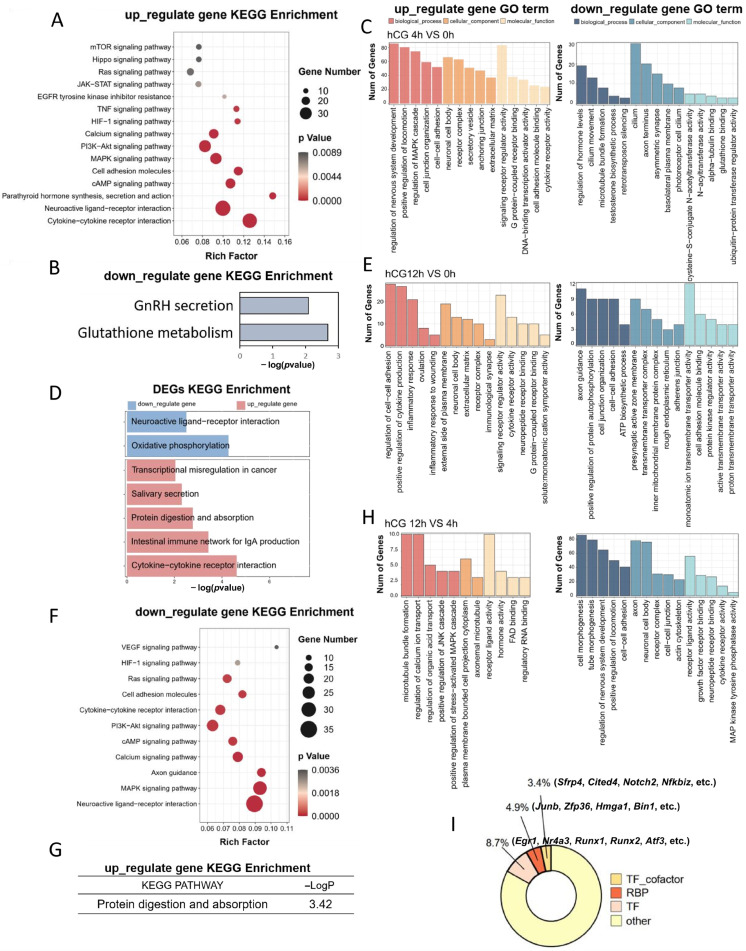
KEGG and GO enrichment analysis of DEGs between 0, 4, and 12 h after hCG induction. (**A**,**B**) KEGG pathway enrichment analysis of DEGs up-regulated (**A**) and down-regulated (**B**) 4 h after hCG treatment. (**C**) GO enrichment analysis of DEGs 4 h after hCG treatment. (**D**) KEGG pathway enrichment analysis of DEGs 12 h after hCG treatment. (**E**) GO enrichment analysis of DEGs 12 h after hCG treatment. (**F**–**G**) KEGG enrichment analysis of down-regulated (**F**) and up-regulated (**G**) DEGs 12 h after hCG treatment compared with 4 h. (**H**) KEGG enrichment analysis of DEGs 12 h after hCG treatment compared with 4 h. (**C**,**E**,**H**) Red indicates up-regulated DEGs, blue indicates down-regulated DEGs; dark colors denote biological processes, intermediate colors denote cell localization, and light colors denote molecular functions. (**I**) Functional categorization of up-regulated genes 4 h after hCG treatment.

KEGG analysis of the 0–4 h transition revealed robust activation of the MAPK, PI3K/Akt, mTOR, Hippo, EGFR, Ras, and cAMP signaling pathways, which have been reported to be related to follicular development [46,47,48,49] (Figure 2A,B). GO enrichment analysis demonstrated that up-regulated DEGs were predominantly associated with biological processes related to the regulation of the MAPK cascade, cell junction organization, cell–cell adhesion, the extracellular matrix, signaling receptor regulator activity, transcription factor activity, and cytokine receptor activity (Figure 2C). In contrast, down-regulated genes were enriched in hormone regulation, testosterone biosynthesis, microtubule bundle formation, and ubiquitin-protein transferase regulation (Figure 2C). These enrichment patterns indicate that LH may influence follicular remodeling and CL formation by regulating MGC movement. Comparing 0 h with 12 h, up-regulated genes clustered in cytokine–cytokine-receptor interaction and cancer-like transcriptional programs, whereas down-regulated genes were enriched in oxidative phosphorylation and neuroactive-ligand-receptor interaction pathways (Figure 2D). GO analysis of the up-regulated genes found that their biological processes are enriched in ovulation, including *Adamts1*, *Edn2*, *Ereg*, *Tnfaip6*, *Il6*, *Nr4a3*, *Npr3*, and *Apln*, while the remaining terms involve inflammation-related events (Figure 2E). This is consistent with previous studies showing that inflammatory response is an important characteristic of ovulation [50,51]. Moreover, we found that the 4–12 h down-regulated DEG set largely mirrored the KEGG pathways enriched in the 0–4 h up-regulated set (MAPK, Ras, HIF-1, cAMP, PI3K-Akt signaling pathway; Figure 2F) and the corresponding GO categories (cell adhesion, microtubule dynamics, morphogenesis; Figure 2H), supporting a model in which LH triggers a transient, early transcriptional burst. Conversely, the minor up-regulated cohort at 12 h was enriched only for protein digestion and absorption (Figure 2G).

Consistent with the STEM-defined early transcriptional wave (Figure 1E), we found 4 h LH/hCG up-regulated genes, including 57 transcription factors (8.7%; *Egr1*, *Nr4a3*, *Runx1*, *Runx2*, *Atf3*, etc.), 22 transcriptional co-factors (3.4%; *Sfrp4*, *Cited4*, *Notch2*, *Nfkbiz*), and 32 RNA-binding proteins subject to post-transcriptional modification (4.9%; *Junb*, *Zfp36*, *Hmga1*, *Bin1*) (Figure 2I). This coordinated induction of regulatory genes likely drives the subsequent decline of early-response transcripts, confirming that LH/hCG evokes a precisely timed, transcription factor-mediated program in mouse MGCs.

### 3.3. LH Signaling Induces Dynamic Changes in H3K4me3 and H3K27ac in Mouse MGCs

**Figure 3:**
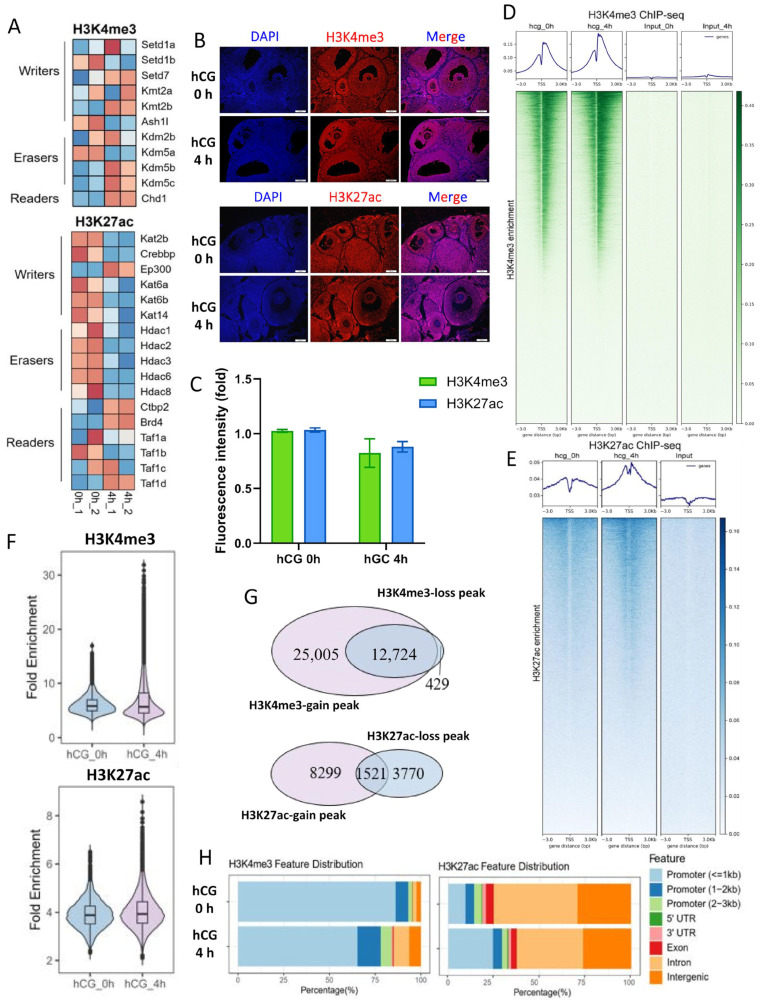
Genome-wide enrichment and redistribution of H3K4me3 and H3K27ac in MGCs at 4 h after hCG treatment. (**A**) Heatmap showing expression of H3K4me3- and H3K27ac-associated modifying enzymes at 0 and 4 h after hCG treatment. (**B**,**C**) Immunohistochemical staining (**B**) and quantification (**C**) of H3K4me3 and H3K27ac in preovulatory follicles at 0 and 4 h after hCG administration (*n* = 3, error bars represent SEM). (**D**,**E**) H3K4me3 (**D**) and H3K27ac (**E**) enrichment around peak center for MGCs at 0 and 4 h after hCG treatment. The upper panels show the average signal profile around detected peak centers (±3 kb). The lower heatmap shows the read density around the peak centers. (**F**) The violin plot shows peak enrichment across samples. (**G**) A Venn diagram of H3K27ac and H3K4me3 ChIP-seq peaks. The numbers indicate the total peaks identified at each time point. (**H**) Stacked bar chart showing the genomic distribution of H3K4me3 and H3K27ac peaks before and after hCG treatment.

It is well established that histone modifications serve as critical switches for gene expression. Transcriptome profiling revealed that the decisive transcriptional switches occur within 4 h of hCG exposure [43,52]. Consistent with this, genes encoding histone-modifying enzymes associated with H3K4me3 and H3K27ac showed marked expression changes (Figure 3A). For example, genes of H3K4me3 demethylase, *Kdm5b*, *Kdm5c*, etc., were significantly up-regulated, and the deacetylating-modifying enzyme genes *Hdac1*, *Hdac2*, and *Hdac3*, etc., in H3K27ac modification were significantly down-regulated.

To determine how these changes affect chromatin, we examined H3K4me3 and H3K27ac levels at 0 h and 4 h after hCG administration. Immunofluorescence staining showed no significant change in the overall abundance of either modification (Figure 3B,C). Re-analysis of public ChIP-seq datasets likewise demonstrated no global difference in normalized signal intensity (Figure 3D,E) or overall peak enrichment (Figure 3F).

We next assessed genome-wide redistribution of these two histone marks before versus after hCG injection. At 4 h after-hCG, H3K27ac gained 8299 and lost 3770 peaks, while H3K4me3 gained 25,005 and lost only 429 peaks (Figure 3G), indicating extensive remodeling. Fragment annotation revealed that >80% of H3K4me3 peaks localized to promoters, whereas H3K27ac occupied only ~30% of promoter regions. Importantly, the genomic distribution of both marks shifted significantly after hCG stimulation (Figure 3H). Together, these results show that LH does not alter the overall abundance of H3K4me3 or H3K27ac, but instead drives large-scale redistribution of these histone marks across the genome, consistent with a rapid chromatin-remodeling response in MGCs.

### 3.4. Integrative Analysis of the Effects of H3K4me3 and H3K27ac Modifications on Transcriptional Activity in Mouse MGCs

**Figure 4:**
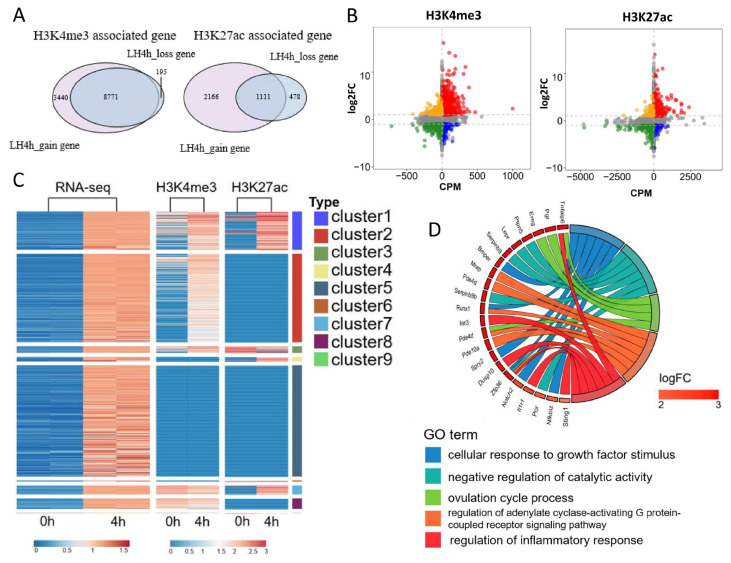
Integrative analysis of histone modification and transcriptome. (**A**) Counts of genes gaining, losing, or maintaining H3K4me3 and H3K27ac after hCG treatment. (**B**) Correlation between mRNA fold change and histone modification enrichment. (**C**) Heatmap of RNA-seq and ChIP-seq relative expression. (**D**) GO enrichment analysis of Cluter1 gene.

To dissect how LH/hCG reshapes the mouse MGC transcriptome, we integrated the analysis of ChIP-seq profiles of H3K4me3 and H3K27ac with matched RNA-seq data. Quantification of histone-mark dynamics showed that H3K4me3 was gained at 3440 genes, lost at 195, and unchanged at 8771; for H3K27ac, 2166 genes gained and 478 lost peaks, whereas 1111 remained static (Figure 4A). Subsequently, we integrated the histone-mark changes with transcriptional output. Most genes fell into the quadrants showing concordant regulation of histone marks and mRNA levels, indicating strong positive correlations between H3K4me3/H3K27ac dynamics and transcriptional change (Figure 4B). Notably, H3K4me3 exhibited a higher proportion of positively correlated genes than H3K27ac, suggesting that H3K4me3 may exert a stronger influence on LH-induced transcriptional activation (Figure 4B).

Furthermore, we clustered the DEGs that were significantly up-regulated at 4 h based on their H3K4me3 and H3K27ac changes, generating nine combined chromatin–transcription clusters (Figure 4C). Cluster 1 contained 109 genes that simultaneously gained both H3K4me3 and H3K27ac and showed a pronounced transcriptional surge, representing the strongest LH-responsive gene cohort (Figure 4C). Functional enrichment linked these rapid-response genes to ovulation, inflammatory signaling, control of catalytic activity, and growth-factor responsiveness, underscoring that the coordinated deposition of H3K4me3 and H3K27ac primes MGCs for the acute ovulatory program (Figure 4D).

### 3.5. H3K4me3 and H3K27ac Affect Transcriptional Activity of Mouse MGCs Through Promoters and Enhancers, Respectively

**Figure 5:**
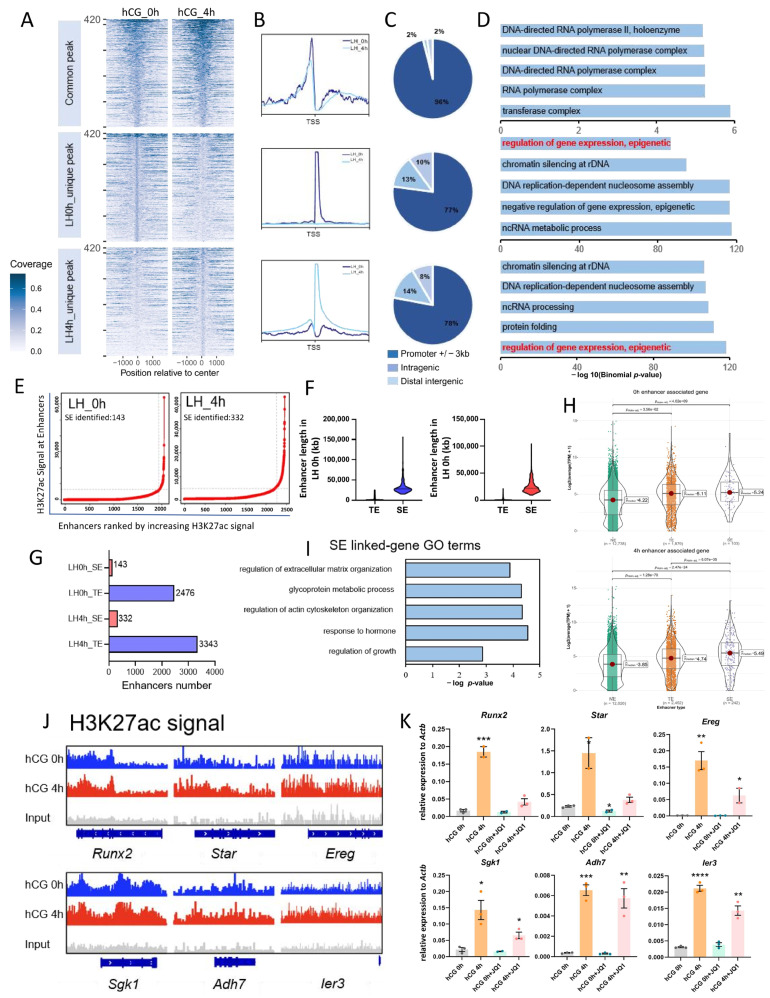
Differential analysis of H3K4me3 peaks and enhancer profiling of H3K27ac. (**A**) Heatmap showing the normalized H3K4me3 signals centered on enriched regions (±1 kb), subdivided according to their dynamic status after hCG treatment. (**B**) Aggregated signal profiles of LH 0 h_unique, LH 4 h_unique, and common peaks. (**C**) Genomic distribution of H3K4me3 peaks before and after the hCG in MGCs. (**D**) Function annotation of genes associated with stage-specific H3K4me3 peaks. (**E**) Identification of enhancers ranked by increasing H3K27ac signal in MGCs at 0 h and 4 h after hCG treatment. The enhancers above the inflection point were defined as original SEs. (**F**,**G**) Comparisons of enhancer length (**F**) and enhancer number (**G**) in MGCs at 0 and 4 h after hCG treatment. (**H**) Comparisons of expression from SE-linked genes, TE-linked genes, and NE-linked genes. (**I**) GO enrichment analysis of SE-linked genes. (**J**) H3K27ac signal profiles in SE-linked and TE-linked genes. (**K**) Expression levels of SE-linked and TE-linked gene mRNA in isolated preovulatory MGCs from mice that were intraperitoneally administered with hCG, with or without JQ-1 (*n* = 3, error bars represent SEM). * *p* < 0.05, ** *p* < 0.01, *** *p* < 0.001, **** *p* < 0.0001 vs. hCG 0 h (*t*-test).

To determine how H3K27ac and H3K4me3 orchestrate the transcriptional activity of mouse MGCs, we divided H3K4me3 fragments into three types: 0 h_unique, 4 h_unique, and common fragments (Figure 5A). Signal quantification confirmed a clear separation of dynamic and static peaks (Figure 5B). Subsequently, we annotated the specific fragments and counted their distribution on the genome, and found that H3K4me3 modification was still mainly located in the promoter region (Figure 5C). Functional analysis using GREAT revealed that stage-specific H3K4me3 peaks were uniformly linked to transcriptional regulation, consistent with its canonical promoter-activating role (Figure 5D).

In contrast, H3K27ac is a canonical enhancer mark [53]. Using ROSE, we perform enhancer analysis on H3K27ac modification at 0 h and 4 h after hCG (Figure 5E). Super-enhancers (SEs) were substantially longer than typical enhancers (TEs) (Figure 5F). We found that SE numbers rose from 143 to 332 after hCG, whereas TEs increased from 2476 to 3343 (Figure 5G). We annotated enhancers and divided genes into three categories: no enhancer (NE)-associated genes, SE-associated genes, and TE-associated genes (Figure 5H). At 0 h, SE-associated genes and TE-associated genes were already significantly higher than those of NE-associated genes, but the transcription levels of SE-associated genes were the same as those of TE-associated genes; whereas at 4 h, SE-associated genes displayed significantly higher induction than TE-associated genes, indicating that enhancer activation, especially SE formation, is a major driver of LH-responsive transcription (Figure 5H). Functional profiling of the 4 h-SE-associated genes highlighted extracellular matrix organization, cytoskeletal dynamics, hormone action, and growth control processes that mirror the rapid follicular remodeling triggered by LH (Figure 5I).

To further verify whether H3K27ac affects gene expression, we blocked the H3K27ac reader BRD4 with JQ-1. IGV snapshots showed that the H3K27ac enrichment signal at LH signaling-induced genes significantly increased 4 h after hCG (Figure 5J). In vivo, JQ-1 abolished the hCG-induced surge of SE- or TE-associated transcripts (Figure 5K). Together, these findings demonstrate that LH/hCG establishes enhancer activity via H3K27ac—particularly SEs—to activate the acute transcriptional program required for ovulation, whereas H3K4me3 primarily operates through promoter regulation.

### 3.6. Disruption of H3K27ac and H3K4me3 Modifications Leads to Impaired Oocyte Maturation and Ovulation

**Figure 6:**
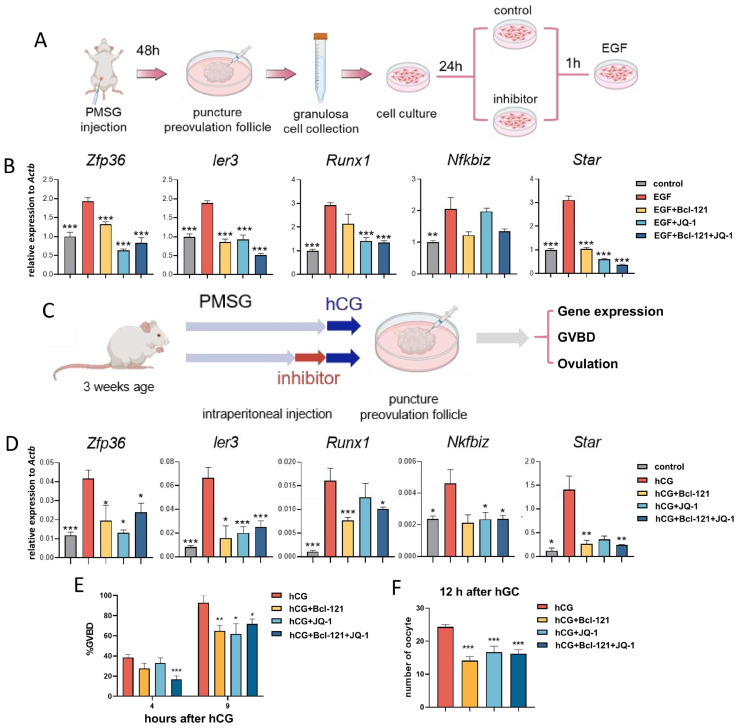
Effect of in vitro or in vivo inhibition of H3K27ac and H3K4me3 on target gene expression. (**A**) Schematic diagram of inhibitor treatment model in vitro. (**B**) Expression levels of cluster1 gene mRNA in in vitro-cultured preovulatory MGCs with inhibitor treatment in vitro. (**C**) Schematic diagram of inhibitor injection model in vivo. (**D**) Expression levels of cluster1 gene mRNA in isolated preovulatory MGCs from mice that were intraperitoneally administered with inhibitors. (**E**) GVBD percentage at 4 h and 9 h after hCG isolated from preovulatory follicles of different inhibitor treatment groups. (**F**) Number of oocytes collected from oviduct at 12 h after hCG in different inhibitor treatment groups. * *p* < 0.05, ** *p* < 0.01, *** *p* < 0.001. *n* = 3, error bars represent SEM; In (**B**), * indicates a significant difference compared with the data from the EGF group (*t*-test). In (**D**–**F**), * indicates a significant difference compared with the data from the hCG group.

To test whether LH regulates oocyte maturation and ovulation through H3K4me3- and H3K27ac-associated pathways, we inhibited SMYD3 (H3K4 methyltransferase) by Bcl-121, and BRD4 (acetyl-histone reader) by JQ-1, in vitro and in vivo. We examined the expression of cluster 1 genes, which are rapidly induced at 4 h. In vitro, preovulatory mouse MGCs were pre-treated with inhibitors for 1 h and stimulated with EGF (a physiological surrogate of LH) (Figure 6A). EGF alone elevated transcript levels within 1 h; single inhibitors partially suppressed this response, while dual blockade kept all targets at lower levels (Figure 6B). We next corroborated these phenotypes in an in vivo model (Figure 6C). Mice were pre-treated for 4 h with vehicle, Bcl-121, JQ-1, or both, followed by hCG injection. RT-qPCR of MGCs collected at 4 h after hCG showed robust induction of target genes in the control group, whereas either inhibitor alone attenuated their up-regulation, and combined treatment almost abolished it (Figure 6D). Thus, coordinated establishment of H3K4me3 and H3K27ac is required for the LH-driven transcriptional surge.

We next assessed the functional contribution of H3K4me3- and H3K27ac-mediated chromatin remodeling to LH-induced oocyte maturation and ovulation. Only combined inhibition significantly reduced GVBD at 4 h, whereas at 9 h, both single and dual treatments impaired oocyte maturation (Figure 6E). Ovulation counts 12 h after hCG revealed a marked reduction in oocyte numbers in all inhibitor groups relative to controls (Figure 6F). Together, these findings indicate that LH signaling instructs MGCs to deposit H3K4me3 and H3K27ac at key loci, thereby enabling the transcriptional program required for timely meiotic resumption, oocyte maturation, and ovulation.

## 4. Discussion

In this study, we profiled the transcriptome dynamics of mouse MGCs at 0, 4, and 12 h after LH/hCG induction and identified distinct temporal patterns of gene expression associated with ovulation. The DEG numbers were lower than previously reported, largely due to stricter filtering (*p*-adj < 0.05), yet the induction of canonical LH-responsive genes, including *Pgr*, *Ptgs2*, *Areg*, *Ereg,* and *Btc*, was highly consistent with prior studies [3,28,43,54,55,56,57]. These results confirm that our dataset faithfully reflects the physiological transcriptional state of MGCs. The strong transcriptional shift at 4 h aligns with the timing of oocyte meiotic resumption and indicates that a major regulatory wave is established early during ovulation.

Functional analyses revealed that up-regulated genes at both 4 and 12 h were enriched for cytokine–cytokine receptor interactions, inflammatory events, and signaling pathways, consistent with the well-established role of a transient, LH-triggered inflammatory-like response during ovulation [12,58,59]. Through transcriptome data analysis, we found that compared with 0 h of LH, whether at 4 h or 12 h, the KEGG terms of up-regulated genes all contained cytokine–cytokine receptor interaction entries, which included genes such as *Cxcr4*, *Ccl5*, *Ccl6*, *Il6*, *Il1b*, and *Csf2*, which is consistent with previous research results. At the same time, the physiological function enrichment results of up-regulated DEGs at 12 h LH included inflammatory response entries, including genes such as *Il1rn*, *Il6*, *Ccr5*, and *Cxcr4*, indicating that there is an inflammatory response-related gene expression network during ovulation [43,60,61]. Notably, *Cxcr4* remained highly expressed throughout, consistent with findings across species [62]. *CXCR4* can modulate ubiquitination-dependent inflammatory duration [7,63,64,65], which indicates that ubiquitination modification in MGCs may play an important role in oocyte maturation and ovulation, which deserves further exploration.

To explore epigenetic mechanisms underlying these rapid transcriptional shifts, we examined LH-dependent changes in histone-modifying enzymes. Numerous genes encoding H3K4me3 and H3K27ac regulators, including *Kdm5b*, *Kdm5c*, and *Hdac1-3*, changed significantly at 4 h, consistent with the notion that epigenetic pathways shape acute follicular responses [4]. Although global H3K4me3 and H3K27ac levels remained stable, ChIP-seq analysis revealed extensive redistribution of both marks. Nearly half of LH-up-regulated genes showed increased H3K4me3, and a substantial proportion showed increased H3K27ac, consistent with their canonical promoter- and enhancer-associated distributions [52,66]. Interestingly, a large gene cluster (cluster 5) exhibited transcriptional activation without detectable histone modification changes, suggesting that transcription factors or other chromatin features also participate in early LH-driven gene induction.

Enhancer analysis further demonstrated that LH robustly expanded both typical enhancers and SEs, particularly those linked to hormone response and growth regulation. Genes essential for steroidogenesis and luteinization, including *Star*, *Cyp11a1*, *Hsd17b*, and *Hsd3b*, acquired new enhancer or SE signatures after LH. These results support a model in which H3K27ac-driven enhancer activation cooperates with promoter-associated H3K4me3 to orchestrate early ovulatory gene expression.

Functional perturbation validated these epigenomic findings. Inhibition of SMYD3 (H3K4 methyltransferase) or BRD4 (H3K27ac reader) attenuated LH/EGF-induced expression of rapidly activated genes, while dual inhibition nearly abolished the response. Disruption of both modifications impaired GVBD at 4 h, reduced oocyte maturation at 9 h, and significantly decreased ovulation at 12 h. These results demonstrate that coordinated promoter and enhancer activation via H3K4me3 and H3K27ac is required for timely meiotic resumption, oocyte maturation, and ovulation.

Overall, this work provides a multi-omics view of LH-induced transcriptional and chromatin remodeling, showing that early ovulatory activation depends not on global histone abundance but on the rapid, locus-specific redistribution of H3K4me3 and H3K27ac. These findings refine our understanding of gonadotropin-regulated chromatin activation and suggest that epigenetic establishment within the early 4 h window may be a critical checkpoint for ovulatory competence and related disorders. Nonetheless, ovulation is a dynamic and prolonged process. Additional time points, spatial transcriptomics [67], single-cell resolution, and comprehensive manipulation of histone writers, erasers, and readers will be essential to further elucidate the full chromatin regulatory network. Moreover, while EGF activation is commonly used to model LH signaling, EGF- and LH-induced epigenomic programs may not be identical, and interpretations from in vitro systems should therefore be made cautiously.

## 5. Conclusions

This study reveals that LH/hCG triggers a rapid and coordinated transcriptional reprogramming in mouse MGCs, driven not by global shifts in histone abundance but by precise, locus-specific redistribution of H3K4me3 and H3K27ac. Promoter-associated H3K4me3 and enhancer-associated H3K27ac, particularly SEs, act together to activate key ovulatory genes within the critical early window after LH stimulation. Functional inhibition of SMYD3 and BRD4 confirms that both histone marks are essential for initiating the transcriptional cascade that supports meiotic resumption, oocyte maturation, and ovulation. These findings provide a mechanistic framework linking gonadotropin signaling to chromatin remodeling and identify early epigenetic establishment as a potential regulatory checkpoint and therapeutic target for ovulation-related disorders.

## Data Availability

The data presented in this study are available in GEO at GSE167938 and GSE165809 (https://www.ncbi.nlm.nih.gov/geo/ (accessed on 3 October 2022)).

## Supplementary Materials

The following supporting information can be downloaded at: https://www.mdpi.com/article/10.3390/cells15010034/s1, Figure S1: Distribution and number of gene expression and PCA analysis of samples. Table S1: Primer sequences.

## Abbreviations

LH	luteinizing hormone
MGCs	mural granulosa cells
SEs	super-enhancers
GCs	granulosa cells
CCs	cumulus cells
LHR	luteinizing hormone receptor
hCG	human chorionic gonadotropin
COC	cumulus–oocyte complex
GVBD	germinal vesicle breakdown
PB1	first polar body
PCOS	polycystic ovary syndrome
CL	corpus luteum
VEGF	vascular endothelial growth factor
EGF	epidermal growth factor
EGFR	EGF receptor
KEGG	Kyoto Encyclopedia of Genes and Genomes
GO	Gene Ontology
CT	threshold cycle
TPM	transcripts per kilobase per million mapped reads
DEGs	differentially expressed genes
STEM	Short Time-series Expression Miner
ROSE	Rank Ordering of Super-Enhancers
TEs	typical enhancers
BSA	bovine serum albumin
ANOVA	one-way analysis of variance
NE	no enhancer
