# Supplementary material for: Ovulatory Signal-Driven H3K4me3 and H3K27ac Remodeling in Mural Granulosa Cells Orchestrates Oocyte Maturation and Ovulation
Source: Cells. 2025 Dec 24;15(1):34. doi: 10.3390/cells15010034 (PMC12785594; doi:10.3390/cells15010034)
Supplement: Supplementary file 1 [file cells-15-00034-s001.zip › Figure S1.pdf]

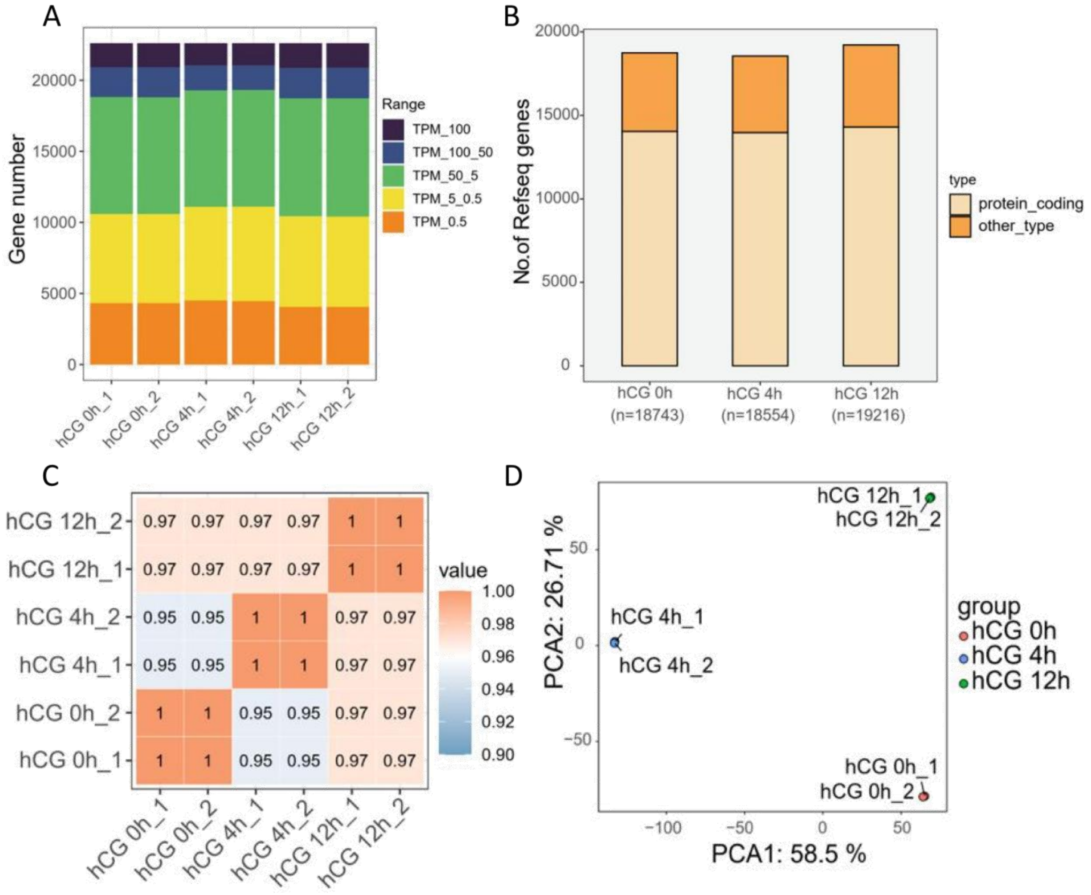

Figure S1. Distribution and number of gene expression and PCA analysis of samples. (A) Accumulation map of gene expression distribution in different samples; Each column represents a sample. (B) Statistics and classification of the number of genes detected in different samples. (C) Pearson's correlation analysis of samples at different time points after hCG administration. (D) The principal component analysis (PCA) of samples at different time points after hCG administration.
