# Supplementary material for: Ovulatory Signal-Driven H3K4me3 and H3K27ac Remodeling in Mural Granulosa Cells Orchestrates Oocyte Maturation and Ovulation
Source: Cells. 2025 Dec 24;15(1):34. doi: 10.3390/cells15010034 (PMC12785594; doi:10.3390/cells15010034)
Supplement: Supplementary file 1 [file cells-15-00034-s001.zip › Table S1.pdf]

Table S1: Primer Sequences.

Primer	Sequences (5′-3′)	Application
Zfp36-F	CGAGAGCCTCCAGTCGATGAG	RT-qPCR
Zfp36-R	GGATGGAGTCCGAGTTTATGTTCC	RT-qPCR
Adh7-F	GCAAAGCGGCTGTCCTATG	RT-qPCR
Adh7-R	GCCAAAATCTTAACACGGACTTC	RT-qPCR
Sgk1-F	GAGCCGGAGCTTATGAACG	RT-qPCR
Sgk1-R	AGTGAAAGTCGGAGGGTTTGG	RT-qPCR
Runx2-F	GACTGTGGTTACCGTCATGGC	RT-qPCR
Runx2-R	ACTTGGTTTTTCATAACAGCGGA	RT-qPCR
Nfkbiz-F	TGCTACACATCCGAAGCAACA	RT-qPCR
Nfkbiz-R	CACTGCACTCTTCAGGTCTGT	RT-qPCR
ler3-F	CAGCCGAAGGGTGCTCTAC	RT-qPCR
ler3-R	AGCCATCAAAATCTGGCAGAAG	RT-qPCR
Runx1-F	TGGTGGAGGTACTAGCTGACC	RT-qPCR
Runx1-R	CGAGTAGTTTTCATCGTTGCCTG	RT-qPCR
Star-F	CCGGGTGGATGGGTCAA	RT-qPCR
Star-R	CACCTCTCCCTGCTGGATG	RT-qPCR
Ereg-F	AACTCAGGAACAATTTACGTCTCTG	RT-qPCR
Ereg-R	GCTTTGGTTCTCAGTATAGAGAGAGA	RT-qPCR
